# DLS 5.0 - The Biomechanical Effects of Dynamic Locking Screws

**DOI:** 10.1371/journal.pone.0091933

**Published:** 2014-04-10

**Authors:** Stefan Döbele, Michael Gardner, Steffen Schröter, Dankward Höntzsch, Ulrich Stöckle, Thomas Freude

**Affiliations:** 1 BG Trauma Center, Eberhard Karls Universitaet Tuebingen, Tübingen, Germany; 2 Department of Orthopaedic Surgery, Washington University School of Medicine, St. Louis, Missouri, United States of America; Van Andel Institute, United States of America

## Abstract

**Introduction:**

Indirect reduction of dia-/metaphyseal fractures with minimally invasive implant application bridges the fracture zone in order to protect the soft-tissue and blood supply. The goal of this fixation strategy is to allow stable motion at the fracture site to achieve indirect bone healing with callus formation. However, concerns have arisen that the high axial stiffness and eccentric position of locked plating constructs may suppress interfragmentary motion and callus formation, particularly under the plate. The reason for this is an asymmetric fracture movement. The biological need for sufficient callus formation and secondary bone healing is three-dimensional micro movement in the fracture zone. The DLS was designed to allow for increased fracture site motion. The purpose of the current study was to determine the biomechanical effect of the DLS_5.0.

**Methods:**

Twelve surrogate bone models were used for analyzing the characteristics of the DLS_5.0. The axial stiffness and the interfragmentary motion of locked plating constructs with DLS were compared to conventional constructs with Locking Head Screws (LS_5.0). A quasi-static axial load of 0 to 2.5 kN was applied. Relative motion was measured.

**Results:**

The dynamic system showed a biphasic axial stiffness distribution and provided a significant reduction of the initial axial stiffness of 74.4%. Additionally, the interfragmentary motion at the near cortex increased significantly from 0.033 mm to 0.210 mm (at 200N).

**Conclusions:**

The DLS may ultimately be an improvement over the angular stable plate osteosynthesis. The advantages of the angular stability are not only preserved but even supplemented by a dynamic element which leads to homogenous fracture movement and to a potentially uniform callus distribution.

## Introduction

In recent years, extramedullary internal fixation techniques of long bone fractures have evolved from very rigid constructs to more flexible internal fixation constructs [1]–[3]. This evolution was possible with the introduction of locking plates, as angular stable screw anchorage in the plate eliminated the need for plate compression against the bone [2]. Contrary to absolute stability with primary bone healing, the aim of a less rigid fixation is the stimulation of fracture healing by callus formation [3]. Terms and definitions like “biological plate osteosynthesis” and “bridge plate technique” were introduced and established [1], [3], [4]. The basic principle of both methods is a minimally invasive reduction and fixation technique preserving fracture vascularity [1]–[6].

**Figure 1:**
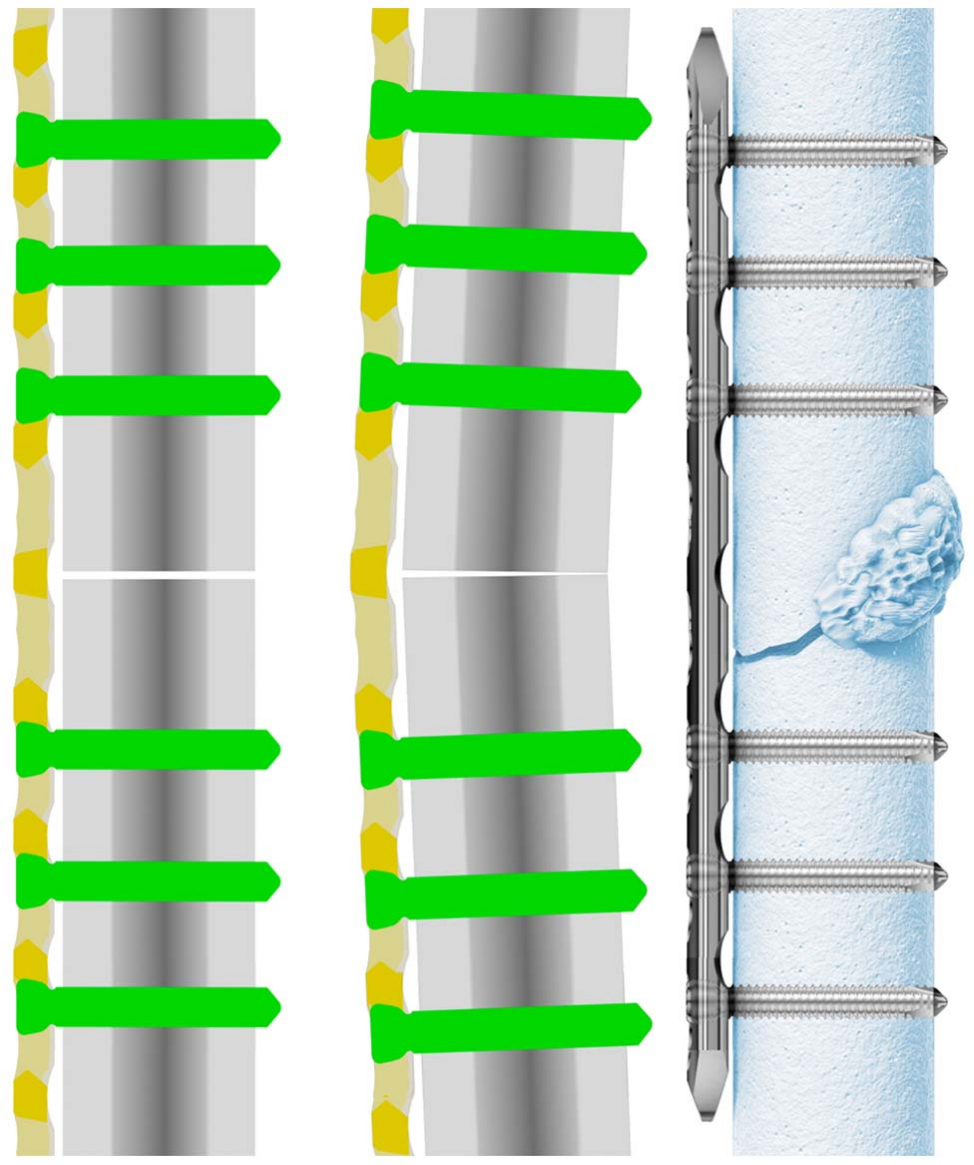
Osteosynthesis with Locking Head Screws without and with axial load and callus formation only at the trans cortex.

Recent studies have confirmed that axial stiffness of modern locked plating constructs is in some cases too high [3], [6]–[8] which is based on the angular stable plate-screw interface in locked plates. The majority of the fracture motion occurs through bending of the plate under axial load resulting in an asymmetric fracture motion, with greater motion at the far cortex than at the near cortex [8]. This may suppress callus formation, particularly at the near cortex (fig. 1) [5]–[13].

**Figure 2:**
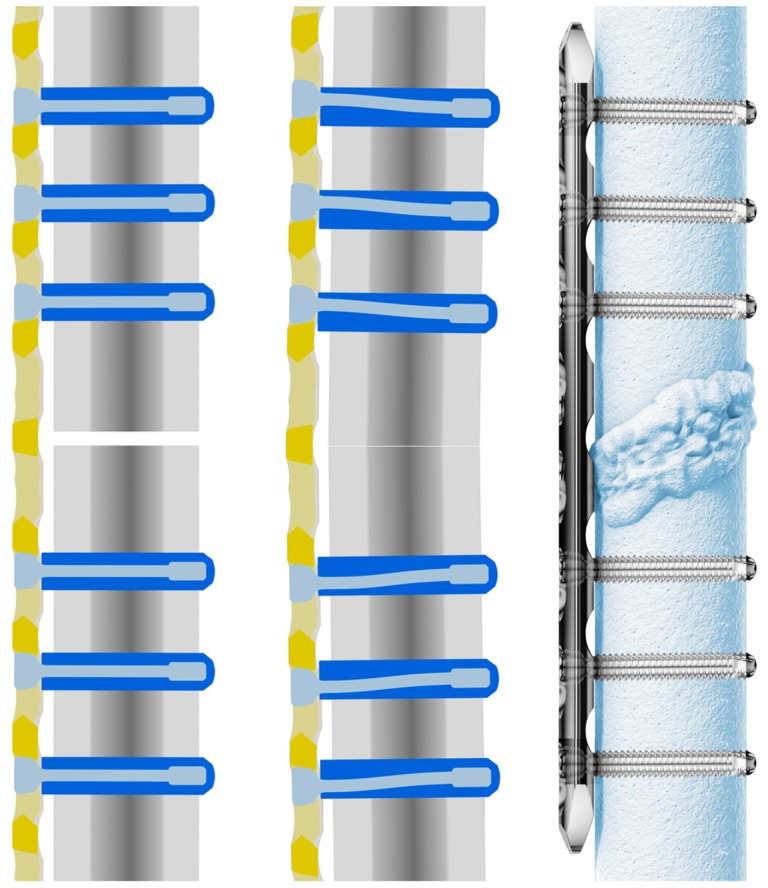
Osteosynthesis with DLS without and with axial load and circumferential callus formation.

Several techniques have been described to decrease the stiffness of a locked plating construct [5], [6], [8], [14]. With the introduction of the 3.7 mm Dynamic Locking Screw (DLS 3.7) in 2010, it was demonstrated that the axial stiffness of a locking plate construct was decreased and the interfragmentary motion of the far cortex was increased, while still providing adequate mechanical stability. These changes result in a more symmetrical fracture motion (fig. 2) [8].

**Figure 3:**
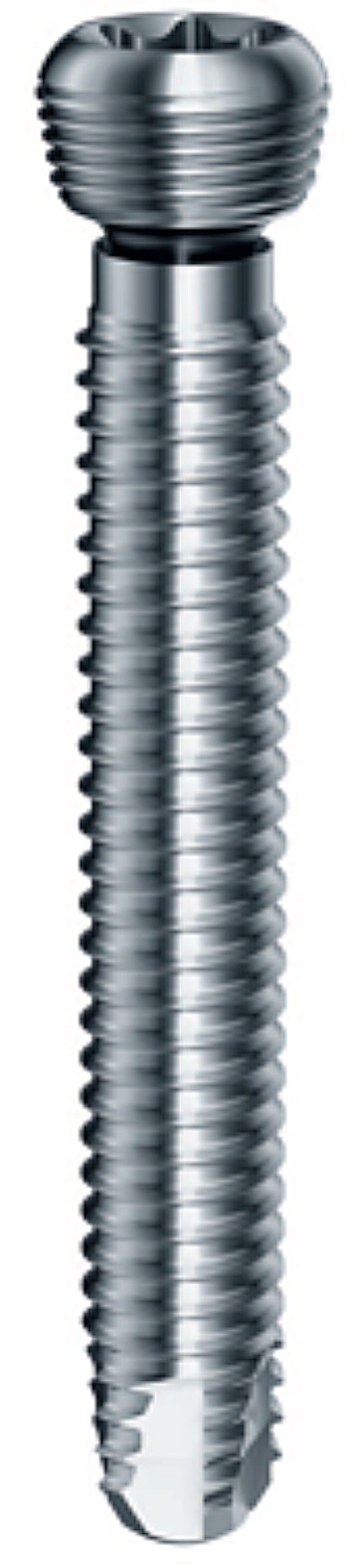
DLS – Dynamic Locking Screw with PIN-Sleeve-Design.

**Figure 4:**
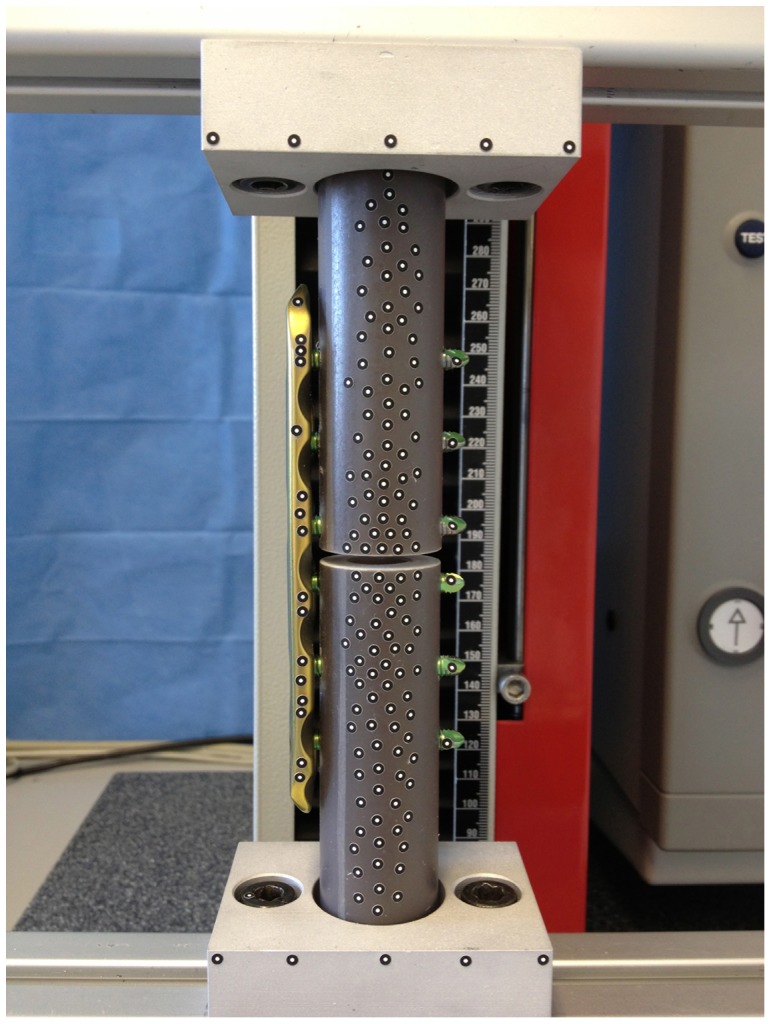
Specimens had a diameter of 27's modulus of 16.7 GPa. Passive markers were attached to both cylinders and the LCP to perform the measurement with the optical motion analysis system.

**Figure 5:**
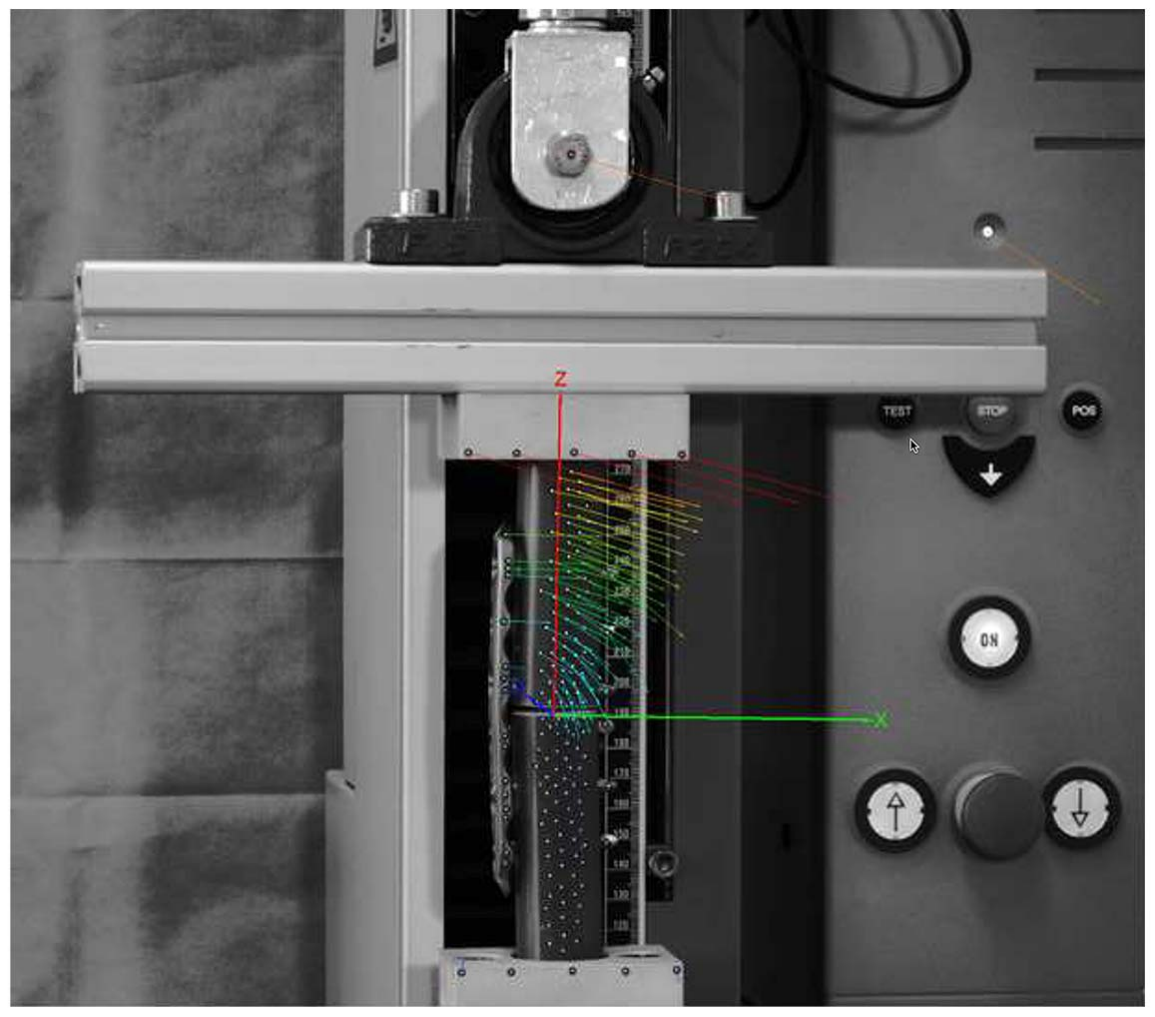
Test set-up. Axial load was applied by a universal testing machine. 3D-motion at the fracture gap was measured with an optical motion analysis system and visualized by vectors. Raw data of the 3D-motion of every passive marker was analyzed using the software MATLAB.

The aim of the following study was to determine the biomechanical properties of large fragment 5.0 mm Dynamic Locking Screw (fig. 3, Synthes, Oberdorf, Switzerland) constructs. To achieve this, we used standardized constructs (fig. 4) with Dynamic Locking Screws (DLS) and standard Locking Head Screws (LS) to assess differences in *i*) axial stiffness and *ii*) interfragmentary motion (fig. 5).

## Materials and Methods

### 1. Specimens

Twelve surrogate bone models were obtained and used for the testing (fig. 4). Two cylinders with a diameter of 27 mm, a wall thickness of 7 mm, a length of 95 mm and a Young's modulus of 16.7 GPa (Sawbones #3403; Pacific Research Laboratories, Vashon, Washington) were used to create a simplified transverse fracture model, according to Bottlang et al. [5]. The holes for the screws were inserted in the cylinders with a computer controlled milling machine. The two cylinders were fixed together using a 6-holes titanium locking compression plates (#426.561 LCP broad 4.5/5.0, L = 116 mm, TiCP, Synthes, Oberdorf, Switzerland). In the first six specimens, DLS (#09.223.038S, 5.0 mm, self-tapping, L38 mm, CoCrMo, Synthes, Oberdorf, Switzerland) screws were tested, while in the second six specimens LS ((#412.213, 5.0 mm, self-tapping, L38 mm, Ti Al6 Nb7, Synthes, Oberdorf, Switzerland) were used. Plates were fixed with three screws per cylinder, which were placed in the first, second and third hole from the fracture site. The fracture gap was 3 mm wide. All screws were tightened to 4.0 Nm with the plate at 2 mm distance from the cylinder surface.

These locked plating constructs, 6 with LS and 6 with DLS, were tested while applying a force-controlled, quasi-static axial load of 0 N to 2.5 kN at 10 mm/min using a mono-axial material testing device (Zwickiline 2.5 kN; Zwick, Ulm, Germany). In this force range (0–2.5 kN), no plastic deformity of the implants was observed. The three-dimensional motion of each osteosynthesis was measured using the optical motion analysis system PONTOS 5M (GOM, Braunschweig, Germany). Using an interface, the force measured by the load cell (testing machine) could be transferred to the optical motion system and processed by the software of the optical motion system. The axial stiffness was calculated using the applied force and the corresponding deviation in the z-axis. Furthermore, motion near the plate (cis-cortex) and far away from the plate (trans-cortex) was calculated. The data was analyzed with Matlab (MathWorks, USA).

### 2. DLS

The DLS is a new generation of locking screws that allows the surgeon to control the rigidity of plating constructs. The DLS pin-sleeve design combines locking technology with dynamic motion — the threaded sleeve anchors the DLS in the bone, while the pin locks the DLS with its standardized threaded head into Synthes locking plates (fig. 3). The play between the sleeve and the pin determines the amount of motion induced in the fracture gap. Cobalt chromium molybdenum alloy (CoCrMo) ensures the mechanical stability of DLS, while encouraging less ongrowth and facilitating screw removal – similar to stainless steel screws. DLS is fully compatible with all Synthes' titanium and stainless steel locking plates.

### 3. Optical motion analysis system

For measuring the 3D fracture motion we used the optical analysis system PONTOS 5 M (GOM - Optical Measuring Techniques, Braunschweig, Germany). The system consists of two CCD (Charge-coupled device) cameras. For the detection of the motion passive markers are required. The points are recorded and tracked by the PONTOS software (fig. 5). The PONTOS 5 M system was set up and calibrated for a measurement volume of 350×280×280 mm according to the manufacturer's documentation. The geometrical setup as well as the optical distortion factors of the lenses were accounted for in the calibration procedure. The frame rate was 4 Hz. White self-adhesive dots with a diameter of 2 mm were used as passive motion markers, with at least 3 points per object. For a higher accuracy, we used about 400 passive markers per specimen. The high number of points allowed fitting two cylindrical geometry elements, which represented the physical cylindrical fracture fragments. The six degrees of freedom motion could, therefore, be directly represented at the location in the center of the fracture gap for each fragment. Relative motion in all six degrees of freedom was analyzed (fig. 5).

### 4. Statistical analysis

All results were analyzed with a commercial statistical software package (GraphPad Prism, El Camino Real, LaJolla, USA, Version 5.01, t-test, p≤0,05).

## Results

**Figure 6:**
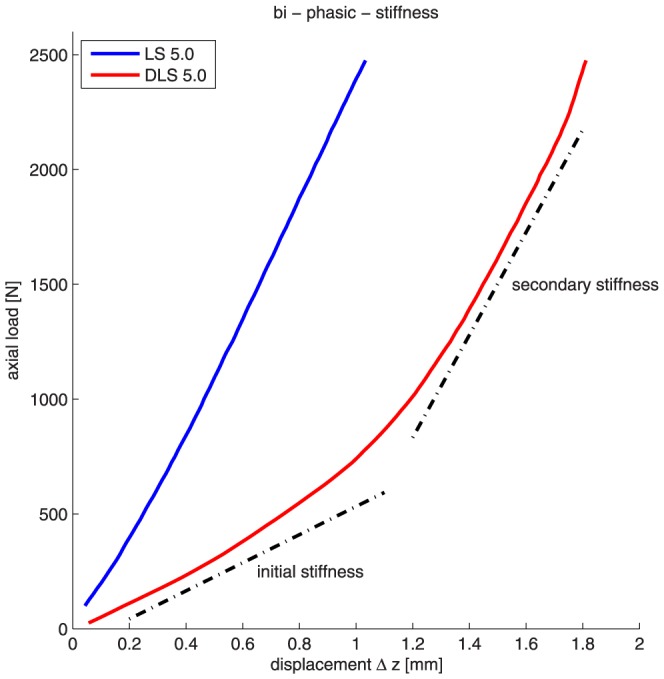
In comparison to the LS, the DLS showed a biphasic stiffness distribution with an initial and a secondary stiffness. The initial axial stiffness of DLS was 612.4/mm and secondary stiffness was 2301.9 N/mm. The mean LS axial stiffness was 2394.9 N/mm.

In comparison to the LS, the DLS showed a biphasic stiffness distribution with distinct initial - and secondary stiffnesses. As shown in figure 6, the initial axial stiffness of the DLS group was 612.4 N/mm and secondary stiffness was 2301.9 N/mm. In the LS group the axial stiffness was generally constant at a mean of 2394.9 N/mm throughout stiffness testing (monophasic stiffness distribution). In the DLS group, the initial stiffness appeared to be a result of the dynamic phase of the screw at low loads. At higher loads, as soon as the pin of the screw has displaced the maximum distance within the sheath, there was a sharp increase stiffness. After the increase there was a secondary stiffness curve, which was similar to the LS. The initial stiffness of the DLS was 74.4% lower than the stiffness of LS. The secondary stiffness of the dynamic construct was 3.4% lower than the stiffness from the non-dynamic construct.

**Figure 7:**
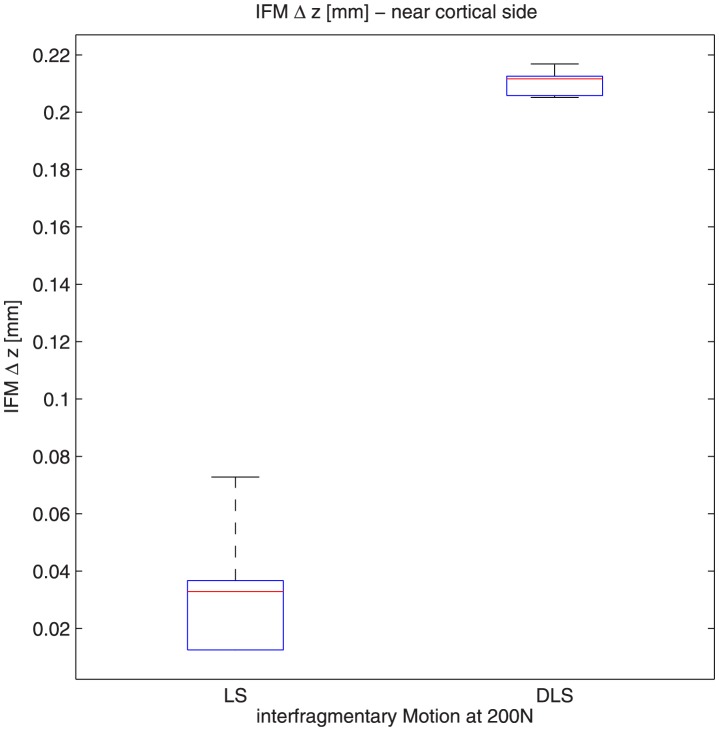
With application of a 200(0.210 mm; SD ± 0,004 mm) (compared to the LS group (0.033 mm; SD ± 0.02 mm).

Interfragmentary motion was significantly greater at the near cortex (fig. 7+8) with the DLS compared to the LS (0.089 mm; SD ± 0,03 mm vs 0.491 mm; SD ± 0,008 mm, respectively, p<0.001) under a 500 N load. At an axial load of 200 N, the interfragmentary motion at the near cortex was seven-fold greater with the DLS (0.210 mm; SD ± 0,004 mm) compared to the LS (0.033 mm; SD ± 0.02 mm) (p<0.001) (fig. 7).

**Figure 8:**
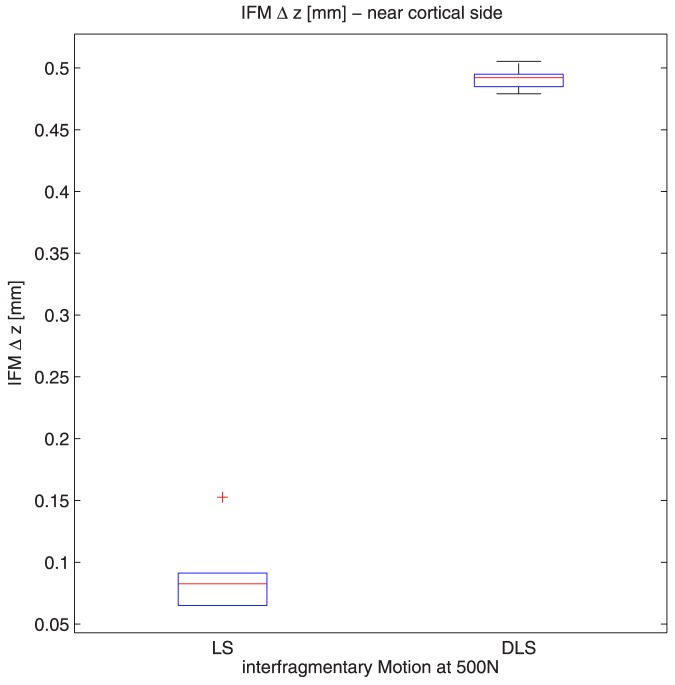
With application of a 500(0.49 mm; SD ± 0,008 mm) compared to the LS group (0.089 mm; SD ± 0,03 mm).

## Discussion

Delayed healing of long bone fractures remains a clinically significant problem. The recent popularity of locking plates has potentially contributed to this problem, as these implants are placed eccentrically and are extremely stiff. The goal of developing a new system with a new screw design was to theoretically retain the advantages of the successful locking screw concept by achieving high stability and bicortical screw bone interface. Additionally, however, this new design is intended to alter the overall axial stiffness of the implant, maintain the bending stiffness of proven plate devices, and increase the near cortex micromovement by more symmetrical axial stress distribution. The aim of our biomechanical investigation was to determine if the new dynamic locking screw was able to meet the functional requirements as theorized. A biomechanical *in vitro* study was performed to assess the differences of a dynamic system using the DLS screws in an osteosynthesis construct compared to standard locking screws. We hypothesized that a dynamic system would result in more balanced fracture motion between the near and far cortices, which could theoretically improve callus formation and equalize callus distribution at the fracture site. To test the hypothesis of the current biomechanical study, two main topics were investigated: the axial stiffness and the interfragmentary motion. We found that stiffness was significantly decreased and near cortex motion was significantly increased in dynamic constructs. The physiological consequence of these altered biomechanics (eg, the callus volume and distribution and the mechanical strength) will be addressed in an ongoing *in vivo* animal study.

In comparison to the non-dynamic screws, the dynamic system provided a significant reduction of the initial axial stiffness up to a certain applied axial load on the construct. While applying a load below 400 N, the axial stiffness of LS and DLS diverge from each other. In the range from 400 N to 2.5 kN however, the axial stiffness of both systems converged again (figure 3). In other words, the dynamic effect of the new screw is more distinctive at lower loads and therefore, would be applicable in a clinical application with a partial weight bearing of 25 kg. The pin-sleeve construction was designed in a way that the pin is not only bending as a result of applied axial load on the construct, but that the deformation of the pin is even S-shaped. The length of the pin is always identical and does not depend on the screw length. Therefore the dynamic effect does not depend on the screw length.

After reaching the maximal range of motion limited by pin and sleeve contact, a sharp stiffness increase occurred, which we denoted the “secondary stiffness”. The secondary “axial” stiffness of the DLS was in the same range as the LS. In other words, the characteristics of the DLS equal those of the LS when higher loads are applied.

Concerning patient safety, it can be noted that the positive effects of the DLS on fracture motion can be observed when a partial weight bearing of around 25 kg is maintained by the patient. But when higher loads are applied to the osteosynthesis, the DLS reacts like a conventional locking head screw. Because typical postoperative protocols for plated lower extremity fractures involve six to eight weeks of “toe-touch” weight bearing, our data can be extrapolated to being applicable in a clinical setting.

When a conventional LS is used, the fracture motion on the far cortex is much higher than on the near cortex, because the interfragmentary motion is caused essentially by the bending of the plate [2], [3], [5], [6], [8].

**Figure 9:**
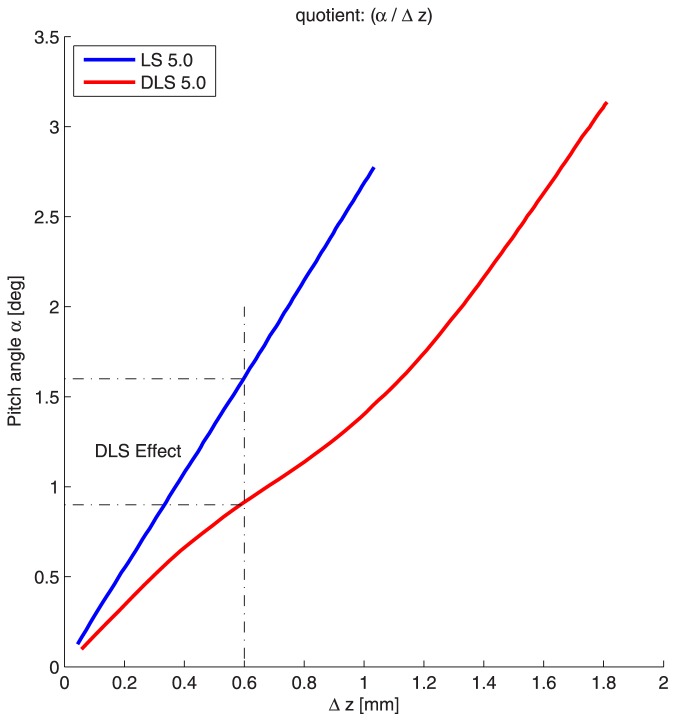
With the same pitch angle of the osteosynthesis plate, interfragmentary motion is higher using the DLS than the LS.

As is shown in figure 6, the DLS enables the same fracture motion while causing a less bending of the plate. This is called the “DLS-Effect” (fig. 9).

The current biomechanical study was performed in a bone surrogate model. This model was chosen because it enabled reproducible data and allowed for the detection of relatively small differences between the dynamic and the standard system. However, this study also had some limitations. As all tests were carried out with artificial bones, it did not reproduce the human biology. Therefore, we did not simulate the effect of osteoporosis on the two systems, and did not analyze the properties of different fracture types. But this was not in focus of the present study, in which the goal was solely to determine the effect of the DLS in an osteosynthesis construct.

Our testing parameters were chosen deliberately to allow comparisons to previous work in the literature. Several previous studies have examined the biomechanics of “dynamic” locked plating systems. [5], [6], [8]. Bottlang et al. evaluated the concept of “far cortical locking” (FCL). [5]. This system also showed a biphasic axial stiffness distribution with an initial and a secondary stiffness. Our findings concurred with those authors, in that more balanced fracture motion at the near and far cortices occurred, the axial stiffness was reduced and the distribution of the interfragmentary motion was more homogeneous [5]. The main difference between the DLS and the FCL is the bone fixation of the screws. While FCL allows only for unicortical fixation at the far cortex, DLS functions more like a standard locking screw, allowing bicortical fixation as the sleeve obtains thread purchase at the near cortex [8]. We believe that the bicortical construct likely improves fixation due to more points of bone-screw contact. Additionally, the dynamic component of the FCL concept relies on the screw shaft toggling against a bone hole at the near cortex, which may cause bone resorption and loss of fixation over time. This is in contrast to the DLS, in which a metal pin toggles against a metal sleeve.

The DLS may ultimately be an improvement over the angular stable plate osteosynthesis. The advantages of the angular stability are not only preserved but even supplemented by a dynamic element which leads to homogenous fracture movement and to a potentially uniform callus distribution. The surgeon has the opportunity to deliberately influence the rigidity of an osteosynthesis through the DLS.
